# Protocol for *in vivo* analysis of muscle function in porcine models for muscular dystrophies

**DOI:** 10.1016/j.xpro.2026.104369

**Published:** 2026-02-12

**Authors:** Hristiyan Hristov, Michaela Blasi, Igor Neves Barbosa, Elisabeth Kemter, Mayuko Kurome, Barbara Kessler, Valeri Zakhartchenko, Nikolai Klymiuk, Michael Stirm, Florian Jaudas, Eckhard Wolf

**Affiliations:** 1 Chair of Molecular Animal Breeding and Biotechnology, Gene Center and Department of Veterinary Sciences, LMU Munich, 81377 Munich, Germany; 2 Center for Innovative Medical Models (CiMM), LMU Munich, 85764 Oberschleißheim, Germany; 3 Human Genome and Stem Cell Research Center (HuG-CELL), University of São Paulo, São Paulo, Brazil; 4 German Center for Child and Adolescent Diseases (DZKJ), 80337 Munich, Germany; 5 Large Animal Models in Cardiovascular Research, Internal Medical Department I, Technical University of Munich, 81675 Munich, Germany; 6 Laboratory for Functional Genome Analysis (LAFUGA), Gene Center, LMU Munich, 81377 Munich, Germany; 7 Interfaculty Center for Endocrine and Cardiovascular Disease Network Modelling and Clinical Transfer (ICONLMU), LMU Munich, 81377 Munich, Germany

**Keywords:** Health Sciences, Genetics, Model Organisms, Biotechnology and bioengineering

## Abstract

Porcine models carrying *DMD* mutations recapitulate Duchenne and Becker muscular dystrophy (DMD and BMD), inherited disorders leading to progressive muscle weakness, with DMD presenting a far more severe and rapidly progressive phenotype. Accurate *in vivo* muscle function assessment is essential for monitoring disease progression and as an efficacy readout for therapeutic intervention. We present a protocol to evaluate muscle strength, dynamics, and fatigue in dystrophic pigs, including design setup, anesthesia, measurement, and data analysis.

For complete details on the use and execution of this protocol, please refer to Blasi et al.^1^

## Highlights

• Standardized protocol for muscle force characterization in a large animal model
• Objective assessment of strength, contraction/relaxation, and fatigue
• Direct electrical stimulation under anesthesia for reproducible results
• Non-lethal workflow enabling longitudinal studies

## Abstract (editor highlights)

Publisher’s note: Undertaking any experimental protocol requires adherence to local institutional guidelines for laboratory safety and ethics.

## Graphical abstract

**Figure:**
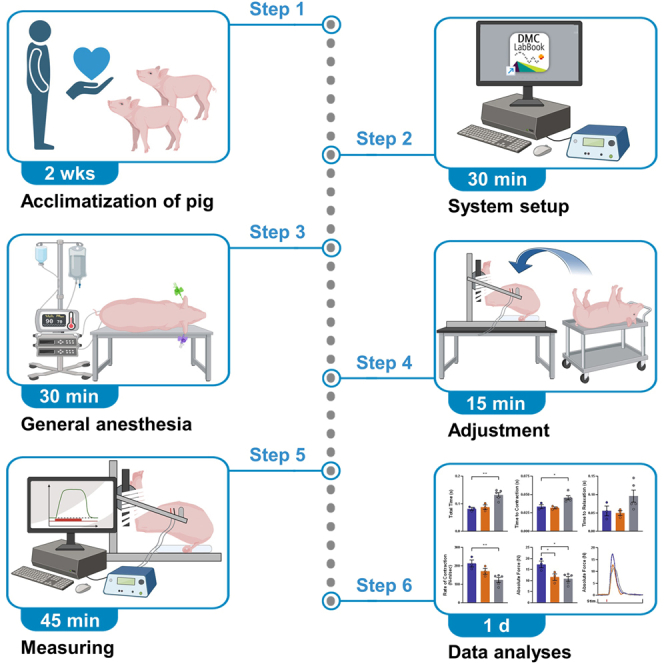

## Before you begin

Duchenne muscular dystrophy (DMD) is an X-linked inherited neuromuscular disorder caused by mutations in the *DMD* gene, leading to a complete loss of functional dystrophin. This results in progressive muscle weakness, loss of ambulation, and ultimately cardiac and respiratory failure.^2^

Animal models are essential for investigating pathomechanisms, diagnostic procedures^3^ and potential therapeutic approaches (^4^; reviewed in^5^). Due to their pathophysiological similarity to humans, pig models offer a particularly suitable system for studying DMD.^6^^,^^7^^,^^8^^,^^9^ *DMDΔ51–52* pigs exhibit a milder disease phenotype with a partially functional dystrophin and clinically resemble Becker muscular dystrophy (BMD).^10^

In this protocol, we describe the use of an isometric muscle force measurement system (Aurora A892 Swine Isometric Footplate Test Device) for the functional characterization of skeletal muscle in genetically modified pig models with specific *DMD* mutations. We examined DMD pigs and BMD pigs, as well as wild-type (WT) controls.

This approach enables quantitative analyses of muscle strength, contraction and relaxation dynamics, and muscular endurance through electrical stimulation of the dorsal flexor muscles of the hind limbs.

### Innovation

We present a standardized and objective protocol for *in vivo* isometric muscle force assessment in genetically modified pig models of two common neuromuscular diseases. Our approach enables the quantification of muscle strength, contraction and relaxation dynamics, and fatigue through electrical stimulation of the hindlimb dorsal flexor muscles. It uses the Aurora A892 Swine Isometric Footplate Test Device, which is adapted to the anatomical characteristics of pigs. Unlike other commonly used tests in rodents, such as grip strength or treadmill running, this technique is performed under general anesthesia and relies on direct stimulation. This eliminates variability due to stress or individual motivation and ensures consistent testing conditions across animals and timepoints, resulting in highly reproducible and comparable data. The workflow is non-lethal and suitable for longitudinal studies, allowing repeated measurements in the same animal to monitor disease progression or therapeutic response over time. While developed for DMD and BMD pigs, the setup is broadly applicable to pig models of other neuromuscular diseases. Compared to existing methods, this setup enhances objectivity, reproducibility, and translational relevance, making it a valuable tool for functional phenotyping in preclinical research.

The protocol was successfully applied in our recent study, marking the first *in vivo* muscle force assessment in pig models for DMD and BMD.^1^

### Institutional permissions

All procedures involving animals complied with the German Animal Welfare Act and were authorized by the Government of Upper Bavaria (approval numbers AZ 55.2–2532.Vet_02-19-195 and AZ 55.2–2532.Vet_02-22-92).

### Pre-experimental acclimatization

**Timing: 2 weeks (may vary depending on model and individual temperament)**

Description of the pre-experimental habituation necessary for minimizing stress and ensuring reliable handling.

1. Acclimate the pigs to the personnel conducting the experiment.
  a. Begin daily interactions at least two weeks prior to the procedure.
  b. Hand-feed the pigs while gently touching the neck and ear region.
  c. Monitor behavioral responses and adjust handling intensity accordingly.

***Note:*** This area corresponds to the later injection site and should be desensitized through repeated gentle contact.

**CRITICAL:** DMD pigs exhibit a severe disease phenotype and are highly stress sensitive. Proper acclimatization minimizes stress responses and improves data reliability.

### Setup of the measurement protocol

**Timing: 30 min**

Setup and calibration guarantee a consistent stimulation and measurement protocol across experimental series.

2. Calibrate the measurement system before starting the study setup.
  a. Confirm calibration using system documentation or visible status indicators.

***Note:*** If calibration cannot be verified, stop the process and contact trained personnel to perform or confirm calibration.

**CRITICAL:** Uncalibrated systems can compromise data integrity. Always verify calibration status before use.

3. Create a new study.
  a. Open the “Create New Study” window via “File > New Study”.
  b. Enter a unique name in the “Name of Study” field.
  c. Provide a brief description of the study in the “Description of Study” field.
    **CRITICAL:** Ensure the study name is unique and descriptive to avoid confusion during later data retrieval.
  d. Add the parameters Animal ID and Body Weight via the dropdown menu or manual entry in the Animal Parameters section.
    i. Assign units where applicable.
    ii. Check the “Fixed” box before clicking the “Add” button.
      ***Note:*** You may also add optional parameters such as Sex, Date of Birth or Treatment Group. For parameters that are not mandatory, you can omit the “Fixed” checkbox.
  e. Specify the muscle group as “Tibialis Anterior” under investigation in the “Muscles section”.
  f. Add experiments to the study using the “Add Experiment to Study” button.
    i. Select “Twitch” and add in the new opening window the Experiment Name “Test Twitch”.
    ii. Check that Tibialis Anterior is selected as the muscle.
    iii. Enter 0.2 ms for the pulse width.
    iv. Select the “Customize Experiment” button.
    v. In the new window that opens, enter the following information: “Timed”, “Time to Next” to 15 s, and “Repeat” to 4.
    vi. Add these settings using the “Commit Changes” button and check them in the table overview that is displayed.
    vii. Add them to the study protocol using the “Save Sequence” button.
    viii. Select “Tetanus”, add the Experiment Name “Test Tetanus”, check the selected muscle and set frequency to 100 Hz and stimulus time to 500 ms.
    ix. After using the “Customize Experiment” button enter and save the following specifications: “Timed”, “Time to Next” to 15 s and “Repeat” to 2.
    x. Select “Twitch” again and configure the settings as described above, but choose a different name: “Measuring Twitch”.
    xi. Select “Tetanus” again, name it “Measuring Tetanus” and set the repetition to 4, otherwise proceed as in the previous step.
    xii. Select “Force Frequency” with amount 8, frequency range 10–120 Hz (ascending), and stimulus time 500 ms.
    xiii. Give the Experiment name “Measuring Force Frequency” and check if the muscle is correctly added. Add it to the study by clicking the “Create Experiment” button.
    xiv. Select “Fatigue” with stimulation frequency 60 Hz, tetanus duration 500 ms, time between tetani 5000 ms and experiment duration 10 min.
    xv. Give the Experiment name “Measuring Fatigue” and check if the muscle is correctly added.
    xvi. Add it to the study by clicking the “Create Experiment” button.
      ***Note:*** The stimulation parameters and protocol settings can be adjusted according to the specific requirements of the experiment. Users should ensure that the selected values align with the physiological characteristics and the goals of the study.
      **CRITICAL:** Incorrect parameter settings may lead to inaccurate measurements, overstimulation, or even muscle damage. It is essential to verify that all stimulation values are appropriate before starting the experiment.
  g. Click Create Study to finalize the setup and return to the main window.

## Key resources table

**Table:** 

REAGENT or RESOURCE	SOURCE	IDENTIFIER
**Chemicals, peptides, and recombinant proteins**		

Azaporc (40 mg/mL)	Serumwerke Bernburg AG	3187
Isotonic saline 0.9 %, 500 mL	B. Braun SE	3200950
Kodan tincture forte uncolored	Schülke & Mayr GmbH	104005
Ursotamin (100 mg/mL)	Serumwerke Bernburg AG	3169
Xylazin (20 mg/mL)	Serumwerke Bernburg AG	3192

**Experimental models: Organisms/strains**		

Domestic pig (German Landrace), *DMD*Δ52	Own breeding; Male, 3,5 months	NA
Domestic pig (German Landrace), *DMD*Δ52-51	Own breeding; Male, 3,5 months	NA
Domestic pig (German Landrace), wild-type	Own breeding; Male, 3,5 months	NA

**Software and algorithms**		

Aurora Scientific Software	Aurora Scientific Inc.	NA
615A Dynamic Muscle Control LabBook and Analysis Software	Aurora Scientific Inc.	NA
GraphPad Prism 10	GraphPad	NA
BioRender	BioRender	NA

**Other**		

Aurora 892A Swine Isometric Footplate Test Device	Aurora Scientific Inc.	NA
Computer with data acquisition card	Aurora Scientific Inc.	NA
Stimulator with constant current mode	Aurora Scientific Inc.	NA
Monopolar EMG needle electrode	Aurora Scientific Inc.	111-725-24TP
Peha-haft – Cohesive conforming bandage, 8 cm x 21 m	Paul Hartmann AG	300071
Sterican zur Blutentnahme G 20 x 1""/ø 0,90 x 25 mm, gelb	B. Braun SE	4657500
Intrafix SafeSet, 180 cm	B. Braun SE	4063000
LifeVet PT Pulsoximeter	Eickemeyer- Medizintechnik für Tierärzte KG	321860
Thermometer	Microlife AG	NA
Disposable razor	Wilkinson Sword GmbH	W302338200
ES compresses 10 cm x 10 cm	Paul Hartmann AG	401835
Leukoplast 2.5 cm x 5 m	BSN medical GmbH	01532–00
Omnifix Luer 5 mL, off-center	B. Braun SE	4616057V
Omnifix Luer 10 mL, off-center	B. Braun SE	4616103V
Omnifix Luer 20 mL, off-center	B. Braun SE	4616205V
Original Perfusor Line PVC, 75 cm, 0.9 x 1.9 mm	B. Braun SE	8722870N
Softaskin, 1.000 mL	B. Braun SE	180217
VasoVet Intravenous catheter, 22 G, 0.9 x 25 mm, blue	B. Braun SE	4269102

## Step-by-step method details

### Device setup

**Timing: 30 min**

Setup of all device components before the experiment ensures stable measurement conditions.

1. Turn on all components of the Aurora 892A Swine Isometric Footplate Test Device at least 30 minutes before testing to allow thermal stabilization: Computer, Stimulator, Transducer system, Analog-digital interface.
2. Open the measurement software.
3. Click the “Change” button under “Study” and select the predefined study template.
4. Click the “Change” button under “Animal” and enter the relevant data for the animal to be measured.

**CRITICAL:** Ensure that the correct study template is selected and all animal data is entered accurately to avoid errors during measurement.

### Sedation and anesthesia setup

**Timing: 30 min**

Anesthesia allows objective evaluation by removing variability caused by stress or motivation.

***Note:*** The animals should be fasted overnight (approximately 8–12 h).

5. Initiate sedation using intramuscular injection in the neck region behind the earbase of 0.2 mL/kg Ketamine (100 mg/mL) and 0.05 mL/kg Azaperone (40 mg/mL).

***Note:*** For volumes larger than 2 mL use a perfusor line and apply the injection slowly (3–5 s).

6. Allow the pig to lie down voluntarily as sedation takes effect.
7. Once the animal is recumbent, place two venous catheters (e.g., ear veins) to enable reliable intravenous access:
  a. One catheter is used for anesthesia deepening.
  b. The second catheter serves for fluid infusion and acts as a backup in case of catheter failure.
8. Deepen anesthesia using 0.1 mL/kg Ketamine (100 mg/mL) and 0.05 mL/kg Xylazine (20 mg/mL) intravenously.
9. Monitor vital signs continuously, including heart rate, oxygen saturation, respiratory rate and body temperature.
10. Ensure the pig reaches a surgical plane of anesthesia before proceeding with positioning.

***Note:*** The choice of drugs and the recommended dose ranges serve as general guidance and should be adjusted at the discretion of the attending veterinarian, taking into account factors such as age, body weight, breed, sex, physiological status, and individual animal response.

**CRITICAL:** Use consistent anesthetic protocols across all animals to avoid variability in neuromuscular responses.

### Positioning and fixation of the pig

**Timing: 5 min**

Biomechanical alignment and fixation of anasthetized pigs provide positional stability during functional testing (Methods Video S1, positioning and fixation).

11. With sufficient personnel, carefully lift and position the anesthetized pig in supine orientation on the surgical table aligned with the footplate apparatus.
12. Position the left hind limb such that the hip, knee, and ankle joints are each aligned at approximately 90° angles (Figure 1).

**Figure 1:**
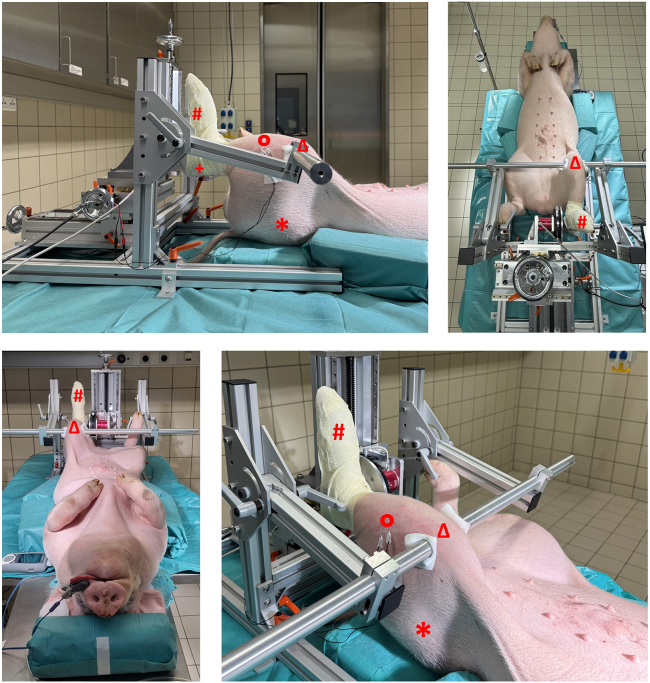
Recommended positioning setup To guarantee accurate biomechanical positioning, the hip (∗), knee (Δ), and hock joints (+) were stabilized at a 90° angle, while the sole was secured to a footplate linked to a highly sensitive torque transducer (#), establishing an ideal physiological setup for generating maximal peak force. Electrodes (Ο) were placed to deliver electrical stimulation to the N. fibularis, with the stimulation current individually fine-tuned to elicit the highest torque response from a single electronic nerve impulse, thereby ensuring targeted activation of the dorsiflexor muscles while preventing engagement of the plantarflexors.
13. Secure the left hind limb to the footplate using cohesive bandages to ensure stable fixation.
14. Stabilize the limb laterally using the adjustable clamping bars to prevent movement during stimulation. If necessary, use positioning aids for stabilization.
15. Fix the head in a neutral position to maintain airway patency and allow unobstructed breathing.

**CRITICAL:** Proper alignment and fixation are essential for reproducible torque measurements and to avoid artifacts due to movement.

Methods Video S1. Positioning and fixation, related to steps 11-28

### Positioning of the electrodes

**Timing: 5 min**

Anatomically consistent electrode placement to maintain reproducibility and prevent stimulation artifacts.

16. Shave the skin area around the fibular head where stimulation electrodes will be placed.
17. Clean the shaved area thoroughly with soap and water to remove any debris.
18. Disinfect the cleaned area using appropriate skin disinfectant.
19. Allow the skin to dry completely before proceeding.
20. Insert the stimulation electrodes percutaneously through the skin at the dorsolateral region of the lower hindlimb, approximately 2-3 cm distal to the fibular head.

**Figure 2:**
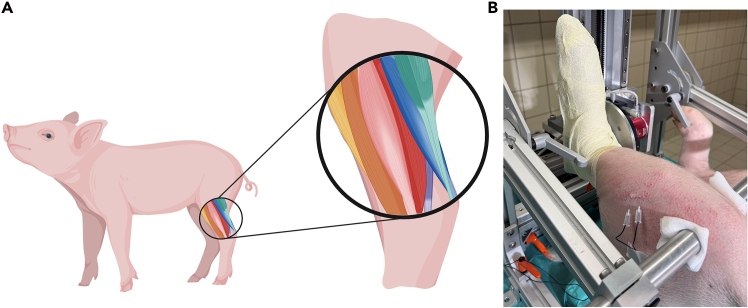
Correct electrode placement (A) Schematic overview of the hindlimb muscles: M. tibialis anterior (yellow); M. fibularis tertius (brown); M. fibularis longus (pink); M. fibularis brevis (red); M. flexor digitorum profundus (purple); M. soleus (blue); M. gastrocnemius (green). (B) Picture of a correct electrode positioning.

***Note:*** This location corresponds to the anatomical region of the tibialis anterior muscle and the course of the fibular nerve. Maintain an inter-electrode distance of approximately 1 cm. Ensure that the electrodes are securely positioned and that insertion depth is consistent across subjects to ensure reproducible stimulation parameters (Figure 2).

21. Fix the electrode cables to the skin using medical-grade tape, leaving a small amount of slack to prevent tension or displacement during movement.

**CRITICAL:** Incorrect placement of the stimulation electrodes may result in activation of unintended muscle groups or complete failure to elicit muscular response. Precise anatomical localization and consistent insertion technique are essential to ensure reproducible stimulation of the target musculature.

***Note:*** If a different stimulation protocol is used, the electrode placement site may be adjusted to target alternative muscle groups. Electrode localization should always be adapted to the specific anatomical and functional goals of the experiment.

### Adjustment of the current intensity

**Timing: 5 min**

Determination of the maximum inducible torque establishes the optimal stimulation intensity for the assay.

22. Open the Instant Stim interface and activate the Live Data Monitor for real-time visualization.
23. Start stimulation at a low current amplitude.
24. Observe the hindlimb for visible movement, confirming activation of the intended muscle group.
25. Simultaneously monitor signal amplitude via the Live Data Monitor.
26. Gradually increase the current in small steps until maximal movement is observed at the live data monitor.
27. Maintain the identified optimal current level for subsequent stimulation.
28. Stop stimulation and allow a 2-minute rest period before continuing.

**CRITICAL:** Starting with a high current may lead to overstimulation or unintended muscle activation. Begin with minimal intensity and adjust gradually.

### Test measurements

**Timing: 5 min**

Characteristic twitch and tetanic contraction slopes confirm proper electrode placement and fixation (Methods Video S2, measurement).

29. Click “Run Experiment” to initiate the twitch stimulation protocol.
30. Observe the resulting force curves representing hindlimb movement on the Live Data Monitor.
31. After twitch testing, allow a rest period of 1 minute.

**Figure 3:**
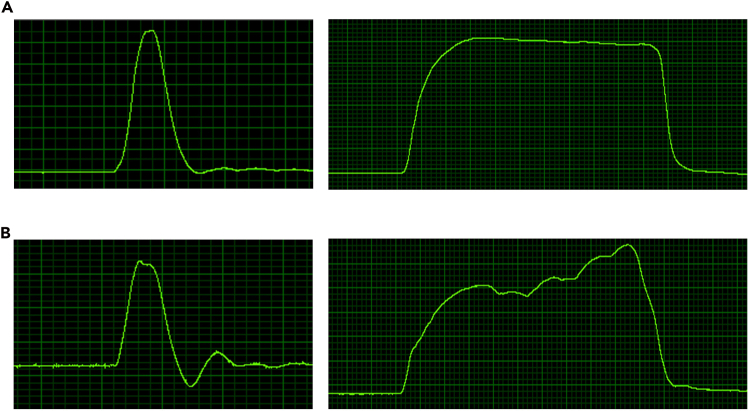
Original slopes (A) Correct trajectories for twitch (left) and tetanus (right). The twitch response shows a single, sharp peak with a steep rise and rapid fall, indicating a properly triggered single muscle contraction. In contrast, the tetanus response displays a clear plateau, reflecting sustained muscle activation. (B) Example of incorrect trajectories, where the characteristic shapes of twitch and tetanus are not clearly distinguishable.

***Note:*** Twitch responses should display a characteristic peak-shaped waveform. If deviations from the expected curve shape are observed, check the proper fixation of the hindlimb and accurate positioning and depth of stimulation electrodes. (Figure 3A left)

Methods Video S2. Measurements, related to steps 29-4

32. Initiate the tetanic contraction test using the designated stimulation protocol.
33. Monitor the force curve during tetanic stimulation.

***Note:*** A stable plateau should be visible, indicating sustained muscle contraction. If the plateau is absent or unstable, reassess fixation, joint angles, and electrode placement. Adjust as needed and repeat the test (Figure 3A, right).

**CRITICAL:** Inconsistent or atypical force curves may indicate mechanical instability or incorrect electrode targeting. These must be corrected before proceeding to ensure valid data acquisition (Figure 3B).

### Measurements (Methods Video S2, measurement)

**Timing: 40 min**

Systematic recording of contractile performance parameters.

34. After a 3-minute rest period following test measurements, begin the main data acquisition sequence.
35. Start with twitch stimulation trials as defined in the study design.
36. Allow a 1-minute pause following twitch measurements.
37. Proceed with tetanic stimulation trials to assess sustained contraction capacity.
38. After a 3-minute rest, initiate the force-frequency stimulation protocol.
39. Allow a 5-minute pause before starting the fatigue stimulation protocol.
40. Upon completion of all stimulation protocols, stop the experiment.

***Note:*** All recorded contractions are automatically saved as extra files.

41. Carefully remove the stimulation electrodes.
42. Depending on the experimental endpoint:
  a. If tissue sampling is planned, maintain anesthesia and proceed with euthanasia under deep anesthesia following approved protocols.
  b. If no sampling is required, allow the animal to recover under controlled observation until fully awake.

**CRITICAL:** Ensure that all stimulation parameters match the predefined study design. Deviations may compromise data comparability and validity.

**CRITICAL:** The protocol must be applied consistently across all animals to ensure data comparability and statistical validity.

**CRITICAL:** Confirm that the animal remains in deep anesthesia throughout the procedure. Spontaneous movements may indicate insufficient anesthesia and can significantly distort measurement outcomes.

### Data analysis

**Timing: 1 day**

Comprehensive data processing workflow encompassing file loading, filtering, cursor adjustment, and standardized result export.

43. Launch the DMA v5.501 software provided by Aurora Scientific.
44. Select the analysis mode:
  a. For single file analysis: Navigate to “File > Open ASI Data File” and select the desired file.
  b. For batch analysis: Click the “High Throughput” tab and choose “Force-Frequency Analysis”, “Force-Time Analysis”, “Fatigue Analysis” or “Position-Time Analysis”.
45. Load data files:
  a. Click “Pick Files” or “Pick Folder” to select all relevant files for one animal.
  b. Files will appear in the Results Table.

***Note:*** Store all files per animal in a dedicated folder to streamline batch analysis.

46. Configure filter and baseline settings:
  a. Enable Filter with Cutoff Frequency: 70 Hz and Falloff Order: 2.
  b. Enable Remove Baseline to subtract baseline from peak force.
  c. Enable Automatic Inversion if negative force values are expected.
47. Set cursor placement method:
  a. Manual: Define Start Cursor and End Cursor manually.
  b. Automatic: Adjust Cursor Threshold, Slope Threshold, Baseline Deviation Notification Threshold, and Baseline Slope Notification Threshold as needed.

***Note:*** If automatic cursor placement is selected, double-check the results to ensure accuracy. If necessary, adjust manually.

48. Run the analysis:
  a. Click Analyze.

***Note:*** Files will be color-coded based on analysis outcome: Green ≙ successful; Red ≙ analysis failed; Orange ≙ baseline deviation error; Yellow ≙ baseline slope error; White ≙ manual cursor placement; Blue ≙ cursor order error

49. Review and correct individual files:
  a. Double-click a row to open the graphical view.
  b. Adjust cursors if needed.
  c. Click Update Cursors to Table to apply changes.
50. Customize output parameters (Table 1):
  a. Click Edit Columns to add, remove, or modify parameters.
  b. Click Save Changes to apply.

**Table 1:** Description of dataset parameters obtained from the measurement

Parameter	Description
Start Cursor/End Cursor (s)	Time points mark the beginning and end of the analyzed contraction event.
Maximum Force	Highest force value recorded during the contraction.
Time to Maximum	Time point at which the maximum force occurs.
Minimum Force	Lowest force value within the data trace.
Time to Minimum	Time point at which the minimum force occurs.
Integration	The area under the force-time curve reflects total work done during contraction.
½ Relaxation Time	Time from peak force (twitch) or end of stimulation (tetanus) to 50% of that force value.
Max Rate of Contraction	The steepest slope of the ascending force curve indicates the rate of contraction.
Time to Max Rate of Contraction	Time point at which the maximum rate of contraction occurs.
Max Rate of Relaxation	The steepest slope of the descending force curve indicates the rate of relaxation.
Time to Max Rate of Relaxation	Time point at which the maximum rate of relaxation occurs.
Starting Baseline	Force value before stimulation begins; used for baseline correction.
Average Force	Mean force value between start and end cursors.
Total Time	Duration between start and end cursors.
Average Rate of Contraction	Mean slope of force increase; can be calculated over full range or specific intervals.
Average Rate of Relaxation	Mean slope of force decrease; can be calculated over full range or specific intervals.
Time to X% Contraction/Relaxation	Time to reach or return to a defined percentage of peak force (e.g. 50%, 95%, 99%, 100%).
Min dx/dt	The minimum rate of change in force reflects the slowest relaxation point.
Custom Time-to-% Parameters	User-defined thresholds for contraction/relaxation timing analysis.
51. Export results:
  a. Click Export Table to Excel or Save Table to ASCII.
  b. Save the file in a structured folder system.

***Note:*** Create a separate Excel file for each animal.

**CRITICAL:** Ensure consistent parameter settings and cursor placement across all samples to maintain data integrity and comparability.

**CRITICAL:** Incorrect settings may lead to inaccurate force measurements and misinterpretation of contraction dynamics.

## Expected outcomes

Based on prior application of this protocol in porcine models of neuromuscular diseases, researchers can expect to obtain detailed and reproducible *in vivo* data on skeletal muscle function, including force output, contraction dynamics, and fatigue resistance. In *DMD*Δ52 pigs, absolute peak force following twitch stimulation typically reaches approximately 62% of WT levels, while tetanic stimulation yields even more pronounced deficits, with force reduced to around 54%. To account for growth retardation and body size differences, all force values are normalized by dividing the measured force by the cube root of body mass. This cube-root scaling approximates the relationship between body mass and linear body dimensions, which are directly relevant for muscle force generation. These normalized values remain significantly reduced in *DMD*Δ52 pigs, confirming the sensitivity and robustness of the measurement approach.

In addition to reduced force, *DMD*Δ52 muscle exhibits marked alterations in contraction kinetics. The total duration of muscle contraction is prolonged, which reflects both an extended contraction phase and a delayed relaxation phase. These abnormalities are most evident following single twitch stimulation. Under tetanic conditions, the high-frequency stimulation partially masks these kinetic differences; however, a significantly slower return to baseline force remains detectable, indicating persistent deficits in muscle relaxation even under sustained activation.

In contrast, *DMD*Δ51–52 pigs show intermediate force values after normalization, reaching approximately 66% of WT levels following twitch stimulation and up to 79% under tetanic conditions. Importantly, contraction and relaxation dynamics in *DMD*Δ51–52 pigs are largely preserved. No significant differences in contraction duration or slope are observed compared to WT. The only notable deviation is a mildly reduced relaxation slope, which appears secondary to the lower peak force rather than indicative of intrinsic kinetic impairment.

During endurance testing, *DMD*Δ52 muscle demonstrates a faster onset of fatigue, reaching 75% of initial peak force after approximately 30 tetanic stimulations. WT and *DMD*Δ51–52 pigs require nearly twice as many stimulations to reach the same threshold, highlighting a clear genotype-dependent difference in fatigue resistance.

These outcomes mirror clinically relevant features of human Duchenne and Becker muscular dystrophy and provide a high-resolution functional readout suitable for longitudinal tracking of disease progression and therapeutic efficacy. The protocol’s sensitivity to both structural and dynamic impairments makes it broadly applicable to other neuromuscular disease models and enhances its translational relevance in preclinical research.

Refer to Blasi et al.^1^ for a full discussion of the outcomes observed in our study.

## Quantification and statistical analysis

The statistical approach employed in this protocol was tailored to the specific aims and outcome measures of the study. Muscle force was quantified using twitch and tetanus recordings, with five consecutive measurements obtained per animal. To mitigate potential adaptation effects, the initial two recordings were treated as premeasurements for checking the correct fixation and electrode positioning and excluded from the final analysis. Furthermore, inclusion of five measurements per contraction type and animal enables averaging and supports the generation of multiple biological replicates, depending on the analytical strategy.

Force normalization was conducted using parameters appropriate to the physiological context. In this study, relative force was calculated by dividing the absolute force by the cube root of body weight, facilitating standardized comparisons across individuals. Depending on the research focus, alternative normalization methods such as adjustment for limb length, muscle mass, or cross-sectional area may be more suitable and can be incorporated accordingly.

To enrich the analysis, additional metrics such as fatigue index, contraction time, and force-frequency relationships can be extracted from raw data traces. These parameters offer deeper insights into muscle physiology and can be integrated into the statistical pipeline based on the scope and objectives of the study.

Data inclusion was determined by signal quality and reproducibility. Measurements exhibiting excessive noise or technical artifacts were excluded to ensure analytical integrity. Statistical analyses were performed using Prism 10 software (GraphPad). To assess data distribution, the Shapiro–Wilk test was applied. As the data followed a Gaussian distribution, statistical significance was determined using a one-way analysis of variance (ANOVA) with Dunnett’s post hoc test for multiple comparisons. A p-value of < 0.05 was considered statistically significant. For studies with non-normally distributed data, non-parametric alternatives such as the Mann-Whitney U test or Kruskal-Wallis test may be appropriate. In longitudinal or multifactorial designs, mixed-effects models or repeated measures ANOVA can be employed to account for intra-subject variability and complex interactions.

## Limitations

This protocol is optimized for juvenile pigs with a body weight of up to approximately 70 kg. Due to the mechanical constraints of the Aurora A892 Swine Isometric Footplate Test Device, including the size and load capacity of the footplate and positioning apparatus, the protocol is not suitable for fully grown adult pigs. Attempting to use the system with heavier animals may result in mechanical failure or damage to the device and may compromise animal safety and data integrity. Furthermore, in neuromuscular diseases with progressive phenotypes, longitudinal studies are inherently limited by the animal’s growth. However, in models of DMD this limitation is less critical. Due to the rapid progression and pronounced phenotype in DMD pigs, the latest measurement of longitudinal studies are conducted at 3.5 months of age, when animals remain within the acceptable weight range for the device.

## Troubleshooting

### Problem 1

DMD pigs are highly sensitive to anesthesia due to their severe neuromuscular and cardiac phenotype. This can lead to respiratory depression, bradycardia, or cardiac arrest, requiring emergency intervention (steps 5–10).

### Potential solution

• Ensure anesthesia is performed by an experienced veterinary team familiar with neuromuscular disease models.
• Monitor vital signs continuously (e.g., heart rate, oxygen saturation, respiration).
• Prepare emergency equipment and resuscitation protocols in advance.

### Problem 2

Electrodes placed incorrectly may activate unintended muscles or produce inconsistent force signals (steps 16–21).

### Potential solution

• Train personnel in pig anatomy and stimulation technique.
• Use anatomical landmarks and palpation to identify the dorsal flexor muscles.
• Always perform test stimulations before starting measurements to confirm correct placement.

### Problem 3

Improper positioning can lead to mechanical artifacts, stress responses, or inaccurate force transmission (steps 11–15).

### Potential solution

• Use supportive fixation devices (e.g., foam pads, straps) to stabilize the animal.
• Ensure the limb is aligned with the footplate and the body is comfortably supported.
• Provide sufficient personnel to assist with positioning and monitoring.

### Problem 4

Inadequate recovery time between stimulations leads to data distortion. Too short intervals between contractions can result in cumulative muscle fatigue, reducing force output and affecting data quality (steps 28, 31, 34, 36, 38 and 39).

### Potential solution

• Ensure sufficient recovery time between stimulation cycles to allow full muscle relaxation.
• Adjust timing parameters to match physiological recovery needs, especially in animals with neuromuscular impairments.
• During longer measurement sessions, monitor muscle tone visually or mechanically to confirm complete relaxation before initiating the next stimulation.

### Problem 5

Data analysis may fail or produce unreliable results due to incorrect cursor placement, signal instability, or software misinterpretation. The DMA software uses a color-coded row system to indicate specific issues. Each color corresponds to a distinct error type that requires targeted correction before finalizing the dataset (steps 43 – 51).

### Potential solution

• Red row - Analysis failed: Ensure that the cursors are positioned precisely at the onset and offset of the contraction signal. Misaligned cursors prevent the software from calculating force parameters.
• Orange row - Baseline deviation too high: Verify baseline stability prior to stimulation. Confirm that the transducer signal is flat and free from drift or pre-contraction fluctuations. Correct baseline deviation.
• Yellow row – Baseline slope drift: Ensure motion artifacts are minimized.
• Blue row - Cursor order error: Adjust cursor sequence manually. The first cursor must mark the contraction onset, and the second cursor the offset. Reversed order will invalidate the analysis.

## Resource availability

### Lead contact

Further information and requests for resources and reagents should be directed to and will be fulfilled by the lead contact, Eckhard Wolf (ewolf@lmu.de).

### Technical contact

Technical questions on executing this protocol should be directed to and will be answered by the technical contact, Eckhard Wolf (ewolf@lmu.de).

### Materials availability

This study did not generate new unique reagents.

### Data and code availability

All data presented are derived from the same experiments reported in Blasi et al.^1^

## Acknowledgments

This study was funded by the Bayerische Forschungsstiftung (AZ 802-08; PDOC-90-15), the Else Kröner-Fresenius-Stiftung (EKFS; 2015_180), and the ForTra gGmbH für Forschungstransfer der EKFS (2018_T20; 2022_EKSE.41). Additional support was provided in part by Leducq Foundation (Network 23CVD01). This project also received partial funding from the Innovative Health Initiative Joint Undertaking (IHI JU) under grant agreement no. 101165643.

Technical support was provided by Chris Rand, MSc (Aurora Scientific Inc.).

The graphical abstract and figures have been created using BioRender.com.

## Author contributions

Conception or design of the work, H.H. and M.B.; data collection, H.H., M.B., and I.N.B.; data analysis and interpretation, H.H., M.B., I.N.B., M.S., F.J., and E.W.; drafting the article, H.H. and M.B.; critical revision of the article, B.K., M.K., V.Z., E.K., N.K., M.S., F.J., and E.W.; final approval of the version to be published, all authors.

## Declaration of interests

The authors declare no competing interests.

## References

[1] Blasi M., Hristov H., Stockl J.B., Kraetzl M., Fiedler S., Kemter E., Kurome M., Kessler B., Cambra J.M., Zakhartchenko V. (2025). Reduced Muscle Force in Dystrophic DMDDelta52 Pigs Is Incompletely Restored by Systemic Transcript Reframing (DMDDelta51-52). J. Cachexia Sarcopenia Muscle.

[2] Duan D., Goemans N., Takeda S., Mercuri E., Aartsma-Rus A. (2021). Duchenne muscular dystrophy. Nat. Rev. Dis. Primers.

[3] Regensburger A.P., Fonteyne L.M., Jüngert J., Wagner A.L., Gerhalter T., Nagel A.M., Heiss R., Flenkenthaler F., Qurashi M., Neurath M.F. (2019). Detection of collagens by multispectral optoacoustic tomography as an imaging biomarker for Duchenne muscular dystrophy. Nat. Med..

[4] Moretti A., Fonteyne L., Giesert F., Hoppmann P., Meier A.B., Bozoglu T., Baehr A., Schneider C.M., Sinnecker D., Klett K. (2020). Somatic gene editing ameliorates skeletal and cardiac muscle failure in pig and human models of Duchenne muscular dystrophy. Nat. Med..

[5] Stirm M., Klymiuk N., Nagashima H., Kupatt C., Wolf E. (2024). Pig models for translational Duchenne muscular dystrophy research. Trends Mol. Med..

[6] Klymiuk N., Blutke A., Graf A., Krause S., Burkhardt K., Wuensch A., Krebs S., Kessler B., Zakhartchenko V., Kurome M. (2013). Dystrophin-deficient pigs provide new insights into the hierarchy of physiological derangements of dystrophic muscle. Hum. Mol. Genet..

[7] Stirm M., Fonteyne L.M., Shashikadze B., Lindner M., Chirivi M., Lange A., Kaufhold C., Mayer C., Medugorac I., Kessler B. (2021). A scalable, clinically severe pig model for Duchenne muscular dystrophy. Dis. Model. Mech..

[8] Frohlich T., Kemter E., Flenkenthaler F., Klymiuk N., Otte K.A., Blutke A., Krause S., Walter M.C., Wanke R., Wolf E., Arnold G.J. (2016). Progressive muscle proteome changes in a clinically relevant pig model of Duchenne muscular dystrophy. Sci. Rep..

[9] Tamiyakul H., Kemter E., Kösters M., Ebner S., Blutke A., Klymiuk N., Flenkenthaler F., Wolf E., Arnold G.J., Fröhlich T. (2020). Progressive Proteome Changes in the Myocardium of a Pig Model for Duchenne Muscular Dystrophy. iScience.

[10] Stirm M., Shashikadze B., Blutke A., Kemter E., Lange A., Stöckl J.B., Jaudas F., Laane L., Kurome M., Keßler B. (2023). Systemic deletion of DMD exon 51 rescues clinically severe Duchenne muscular dystrophy in a pig model lacking DMD exon 52. Proc. Natl. Acad. Sci. USA.

